# Blinded Predictions and Post Hoc Analysis of the Second Solubility Challenge Data: Exploring Training Data and Feature Set Selection for Machine and Deep Learning Models

**DOI:** 10.1021/acs.jcim.2c01189

**Published:** 2023-02-09

**Authors:** Jonathan G. M. Conn, James W. Carter, Justin J. A. Conn, Vigneshwari Subramanian, Andrew Baxter, Ola Engkvist, Antonio Llinas, Ekaterina L. Ratkova, Stephen D. Pickett, James L. McDonagh, David S. Palmer

**Affiliations:** † Department of Pure and Applied Chemistry, University of Strathclyde, Thomas Graham Building, 295 Cathedral Street, Glasgow G1 1XL, U.K.; ‡ Drug Metabolism and Pharmacokinetics, Research and Early Development, Respiratory & Immunology, BioPharmaceuticals R&D, AstraZeneca, Pepparedsleden 1, SE-431 83 Göteborg, Sweden; ¶ GSK Medicines Research Centre, Gunnels Wood Road, Stevenage SG1 2NY, U.K.; § Medicinal Chemistry, Research and Early Development, Cardiovascular, Renal and Metabolism (CVRM), BioPharmaceuticals R&D, AstraZeneca, SE-431 50 Göteborg, Sweden; ∥ Department of Computer Science and Engineering, Chalmers University of Technology, SE-412 96 Göteborg, Sweden; ⊥ Computational Sciences, GlaxoSmithKline R&D Pharmaceuticals, Stevenage SG1 2NY, U.K.; # IBM Research Europe, Hartree Centre, SciTech Daresbury, Warrington, Cheshire WA4 4AD, U.K.

## Abstract

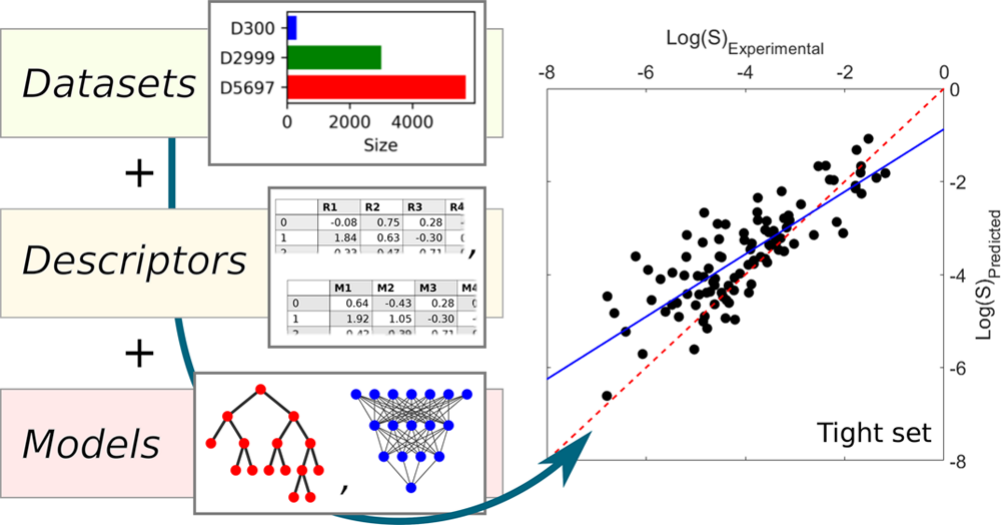

Accurate methods to predict solubility from molecular structure are highly sought after in the chemical sciences. To assess the state of the art, the American Chemical Society organized a “Second Solubility Challenge” in 2019, in which competitors were invited to submit blinded predictions of the solubilities of 132 drug-like molecules. In the first part of this article, we describe the development of two models that were submitted to the Blind Challenge in 2019 but which have not previously been reported. These models were based on computationally inexpensive molecular descriptors and traditional machine learning algorithms and were trained on a relatively small data set of 300 molecules. In the second part of the article, to test the hypothesis that predictions would improve with more advanced algorithms and higher volumes of training data, we compare these original predictions with those made after the deadline using deep learning models trained on larger solubility data sets consisting of 2999 and 5697 molecules. The results show that there are several algorithms that are able to obtain near state-of-the-art performance on the solubility challenge data sets, with the best model, a graph convolutional neural network, resulting in an RMSE of 0.86 log units. Critical analysis of the models reveals systematic differences between the performance of models using certain feature sets and training data sets. The results suggest that careful selection of high quality training data from relevant regions of chemical space is critical for prediction accuracy but that other methodological issues remain problematic for machine learning solubility models, such as the difficulty in modeling complex chemical spaces from sparse training data sets.

## Introduction

Solubility is a fundamental physicochemical property that is important in many aspects of chemistry, particularly in the pharmaceutical industry as it is a key driver in the determination of drug bioavailability.^1,2^ Experimental assays are used to analyze a large number of discovery compounds to screen out problematic low or high solubility compounds.^3^ Empirical solubility determination is strenuous in terms of cost and time, so there is an increasing need for cheaper and faster alternatives for at least the early stages of drug discovery. To meet this need, a large number of computational methods have been developed to complement or, in some cases, replace experimental assays.

The most commonly used predictive methods are data-driven approaches. These statistical models use experimental data to learn a relationship between the physical property of interest (i.e., solubility) and an appropriate computational representation of the molecule. Several hundred such models have previously been published, differing in their choice of molecular encoding, statistical learning algorithm, and training data.^4^ A historical example is the Group Contribution approach, where a (usually) linear relationship is sought between solubility and the number of selected atoms and functional groups in the molecule. A more common approach is to use machine learning algorithms trained on molecular descriptors or fingerprints. Most recent research has been informed by the rapid advances in artificial intelligence being made in other fields. Indeed, a wide-variety of deep learning architectures have been applied to the problem of predicting solubility, including graph-based neural networks,^5−7^ recurrent neural networks,^8^ transformers,^9,10^ message-passing neural networks,^11^ deep belief networks,^12^ and others. Unlike other fields, it is not yet clear that deep learning algorithms offer a significant improvement over traditional machine learning approaches for solubility prediction.^8^ This may partly be due to a lack of accurate experimental solubility measurements for training, although strategies such as transfer learning^8,9^ or multitask learning^13^ may help in some cases.

A well-known advantage of data-driven approaches is that they require very little time to make predictions for single molecules. This makes them suitable for the early stages of drug discovery where there are an incredibly large number of molecules to screen and when the application of predictive models can prioritize within virtual chemical spaces to determine which proposed structures are worth synthetic investment. A significant drawback of data-driven approaches is the requirement for pre-existing high quality experimental data that has been measured under relevant conditions for molecules that are structurally similar to those to be predicted. The solubility data that is available in the published literature varies in quality due to inconsistent methodologies, high experimental errors, varying or undefined experimental conditions, reporting errors, and other issues.^14,15^ Consequently, the volume of reliable data is relatively low and often provides a sparse representation of the relevant chemical space for the compounds being predicted. Reliable models may only be developed when sufficient high quality experimental data are available for compounds from relevant regions of chemical space, which can make it challenging to apply data-driven models either to new compound families or to properties obtained at different conditions (temperatures, solvents, *etc.*). Since data-driven models make predictions from molecular structure, they do not explicitly consider the influence of solid-state polymorphism on solubility. Systematic studies of the solubility differences of known crystalline polymorphs suggest that the average error introduced by this assumption will be less than a factor of 2 in molar solubility for small organic molecules.^16^ Nevertheless, this is an additional confounding factor when many other physiological properties of interest will only be dependent on the intrinsic molecular structure and will be invariant to physical form.

Recently, interest in physics-based solubility prediction has led to several new methods that do not require parametrization against experimental solubility data. The Frenkel group use molecular dynamics simulations to find the conditions where the solution and the solid have the same chemical potential.^17,18^ Kolafa simulates a solute dissolving in a solvent to find the concentration at which equilibrium is reached.^19^ The Anwar group computes the solution density of states from Monte Carlo simulations, yielding a probability distribution function containing two peaks; one is the pure solute, and the mole fraction of the other is the solubility.^20,21^ The Palmer, Mitchell, and Price groups compute solubility from solution free energy, which in turn is obtained from separate calculations of sublimation and hydration free energies by a thermodynamic cycle via the gas phase.^22−24^ Abramov and co-workers use a similar approach with the addition of some empirical parameters.^25^ In principle, these methods have many advantages since they provide a wealth of chemical and thermodynamic data for molecular design, and they are applicable to different solvents, polymorphs, and temperatures without a need for parametrization. However, in practice, their accuracy and computational expense currently limit their practical application to predictions on small systems at low throughput, though that may change in the future.

Over the last 15 years, two blind challenges have been issued by the American Chemical Society to assess the accuracy of solubility prediction methods^26,27^ for small organic solutes. Both challenges have focused on the prediction of intrinsic aqueous solubility at 298 K, which is defined as the concentration of the neutral form of the solute in a saturated aqueous solution at thermodynamic equilibrium at the specified temperature.^22,28^ Although this definition of solubility differs from that commonly measured in some industrial applications, such as the use of so-called “kinetic” solubilities for screening in the pharmaceutical industry, it is an appropriate choice for a prediction challenge where having clearly defined and reliable experimental data is paramount. Moreover, intrinsic aqueous solubilities can be used to predict pH-dependent solubilities and dissolution rates using methods such as the Henderson–Hasselbalch or Noyes-Whitney equations, respectively. In the first solubility challenge issued in 2008,^26^ entrants were provided with a training set of 100 drug-like molecules and asked to submit blinded predictions of the solubility of a further 32 drug-like molecules. Since little information was published regarding the computational methods, nor whether any additional data was employed in training, it is not possible to draw clear conclusions about which methods perform best. Nonetheless, the results confirmed that a root-mean-square error (RMSE) of ∼0.7–1.1 log units was expected from state-of-the-art methods at that time. In the second solubility challenge issued in 2019,^27^ entrants were asked to make predictions of the solubility of 132 drug-like molecules that were divided into two data sets, one comprising 100 molecules considered to have high quality experimental solubility data and one comprising 32 molecules with solubility data with larger experimental errors. Unlike the first solubility challenge in which all experimental data were measured using a single experimental technique, the second challenge used carefully validated experimental data taken from the published literature. Entrants were also invited to submit basic information about the models they employed, which allowed for a more detailed analysis of competing methodologies.

The purpose of this article is two-fold. First, we report two machine learning models that were used to submit blinded predictions to the 2019 solubility challenge, one of which was ranked within the top 10 of all submitted models.^29^ These models were trained on a relatively small data set of 300 molecules. Second, to assess the importance of training data selection, we retrain the models on two larger data sets of 2999 and 5697 molecules and provide a direct comparison to several deep learning algorithms. Critical comparison of these additional sets of models highlights that volume and reliability of experimental data, as well as issues with modeling complex chemical spaces from sparse training data sets, remain problematic, limiting the accuracy of solubility prediction methods.

## Methods

### Experimental Data

Two blinded testing data sets were issued by the second solubility challenge: a “tight” data set comprising 100 molecules with solubility measurements that were consistent across several experimental methods (SD = 0.17 log units) and a “loose” data set comprising 32 molecules with less reliable experimental data (SD = 0.62 log units). To train models to predict the intrinsic aqueous solubility of the tight and loose set molecules, three data sets were compiled from the published literature. “D300” consists of 300 organic and drug-like molecules taken predominantly from the first solubility challenge, supplemented with work published by Llinas, Bergström, and co-workers, as well as from additional sources.^27,30−35^ This data set was used to train the models submitted to the second solubility challenge; the time constraints imposed by the submission deadline combined with the challenges in curating literature solubility data explain the relatively small size of this data set. “D2999” contains the full D300 data set plus additional intrinsic solubility data from Raevsky,^36^ Wang (data set 2),^37^ Louis,^38^ Lovrić,^39^ and Yalkowsky.^40^ “D5697” contains all of the data in D2999 plus additional data taken from AquaSolDB^41^ for molecules which were nonionizable between pH 3–13, based on the OpenEye Quacpac Toolkit (version 2020.2.2)^42^ and OpenBabel (version 3.1.1).^43,44^ D2999 and D5697 comprise 2999 and 5697 molecules, respectively, and these data sets were used to train deep learning models after the second solubility challenge closed. The differing sizes of the three training data sets reflect a trade-off between the reliability of experimental data and the number of data points. We consider the experimental data in D300 to be reliable, but it is a small data set, especially for training machine learning models with multiple free parameters. Conversely, in compiling D5697, we were able to include more molecules but only at the cost of including more experimental measurements of unknown provenance and consequently with unknown variability in experimental methodology.

### Data Set Compilation

The following compound selection and preprocessing rules were used to compile the D300, D2999, and D5697 data sets. SMILES strings were validated by comparing generated structures using PubChem,^45^ CACTUS,^46^ and RDKit.^47^ The specific tautomer defined by the original SMILES was preserved since it was assumed that the original sources contained an appropriate tautomeric form for each compound. Duplicate molecules were identified and removed using InChI strings. RDKit was used to neutralize salts, removing the lowest molecular weight salt component in the process. Other compounds with SMILES strings containing disconnections, indicating multiple components (e.g., solvates), were neglected. The data sets were then filtered to exclude very small (number of heavy atoms < 4), large (molecular weight > 1400), and flexible molecules (number of rotatable bonds > 20) since these were unrepresentative of the solubility challenge data.

## Data Set Analysis

**Figure 1:**
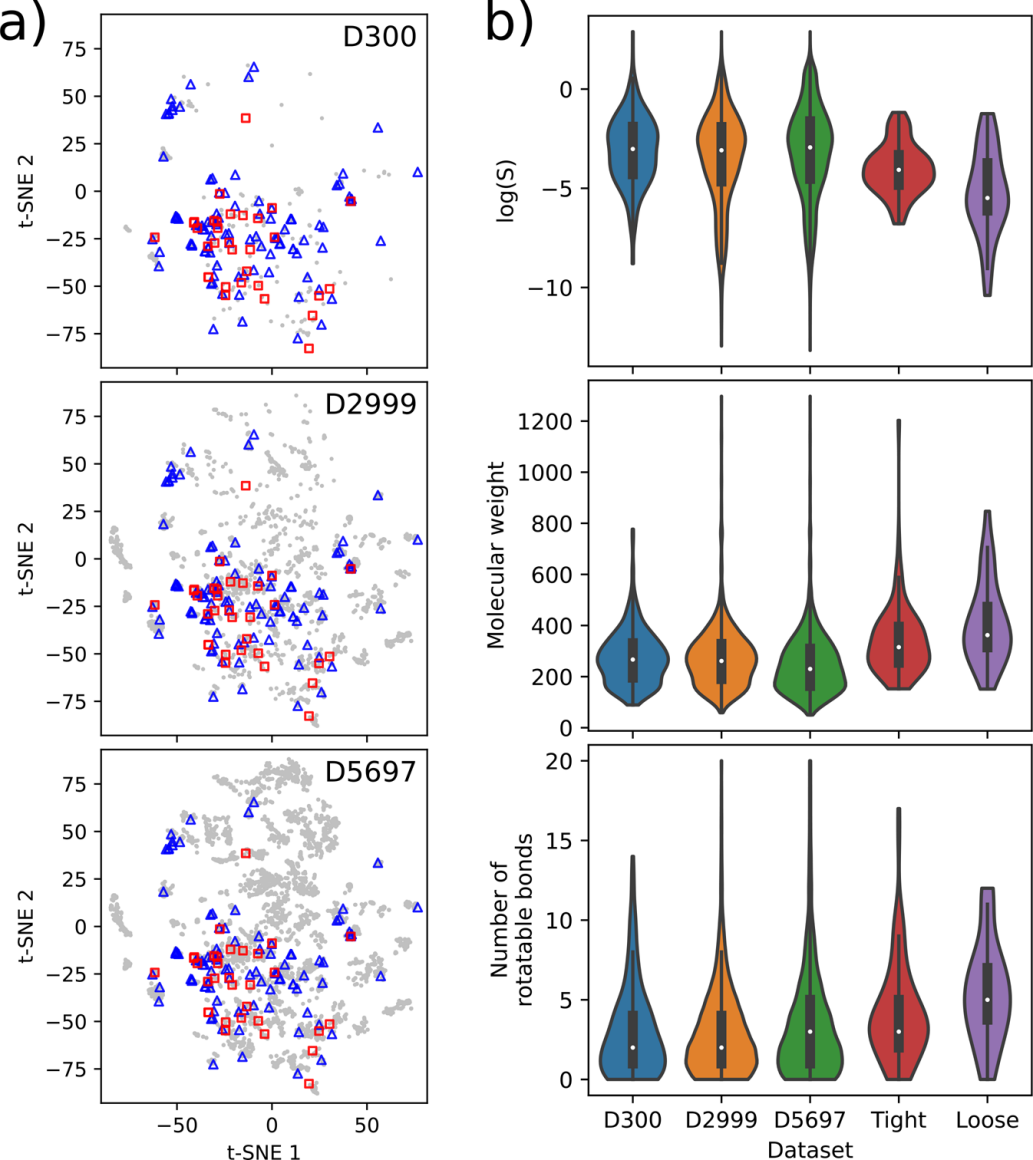
a) t-SNE plots based on RDKit fingerprints for the three training data sets (gray dots) alongside the molecules in the tight (blue triangles) and loose (red squares) testing data sets. b) Violin plots showing the distributions of experimental log(*S*), molecular weight, and number of rotatable bonds for the compounds in the 3 training and 2 testing data sets.

The five data sets (3 training and 2 testing sets) used in this work are summarized and compared in Figure 1. The t-SNE plots (Figure 1.a) illustrate the chemical space spanned by each data set and show that the training and testing sets occupy a similar region. Most of the additional compounds in the larger training sets also occupy a similar space to the testing sets; however, D5697 also contains some more dissimilar compounds due to the inclusion of nonionizable molecules which have fewer or different functional groups than the predominantly neutral ionizable molecules of D2999. The number of unique Murcko scaffolds increases with data set size from 142 (D300), to 798 (D2999), to 1222 (D5697) representing an increase in the coverage of chemical space, which is also evident in the t-SNE plots in Figure 1.a. The violin plots (Figure 1.b) show good overlap in solubility between all five data sets, although the D2999 and D5697 training sets contain a few compounds which extend the ranges to lower solubility values. The range of molecular weights and number of rotatable bonds are also similar across the training sets and reflect the small, drug-like molecules of the testing sets.

**Figure 2:**
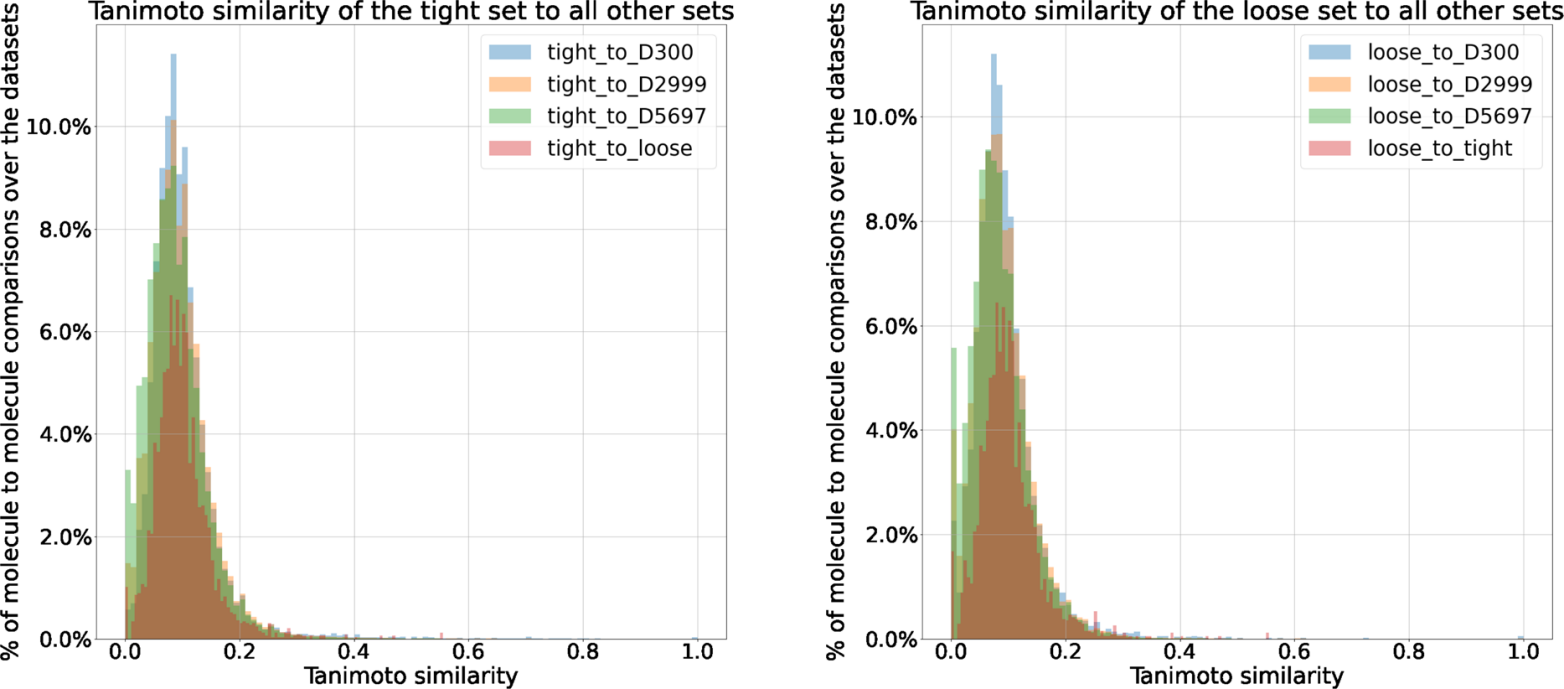
Tanimoto similarity analysis comparing all data sets to the tight set (left) and the loose set (right).

The molecular similarity between our training sets and the testing sets is further illustrated in Figure 2, which displays the Tanimoto analysis between data sets used in the present work. Shown are the distributions of raw set-to-set similarities (i.e., the distribution of pairwise Tanimoto scores between each of the molecules in one data set and each of the molecules in the other data sets) for both the tight and loose sets. The Tanimoto similarity scores are based on Morgan fingerprints and were generated using RDKit (version 2020.09.1b.69). The Morgan fingerprints had a radius of 2 and are 2048 bits in length.

The low Tanimoto similarity scores evident in Figure 2 suggest that the data sets contain a large number of relatively diverse chemical structures. However, there are some molecules with higher scores suggesting some overlap between the training and testing sets in terms of structural similarity.

**Figure 3:**
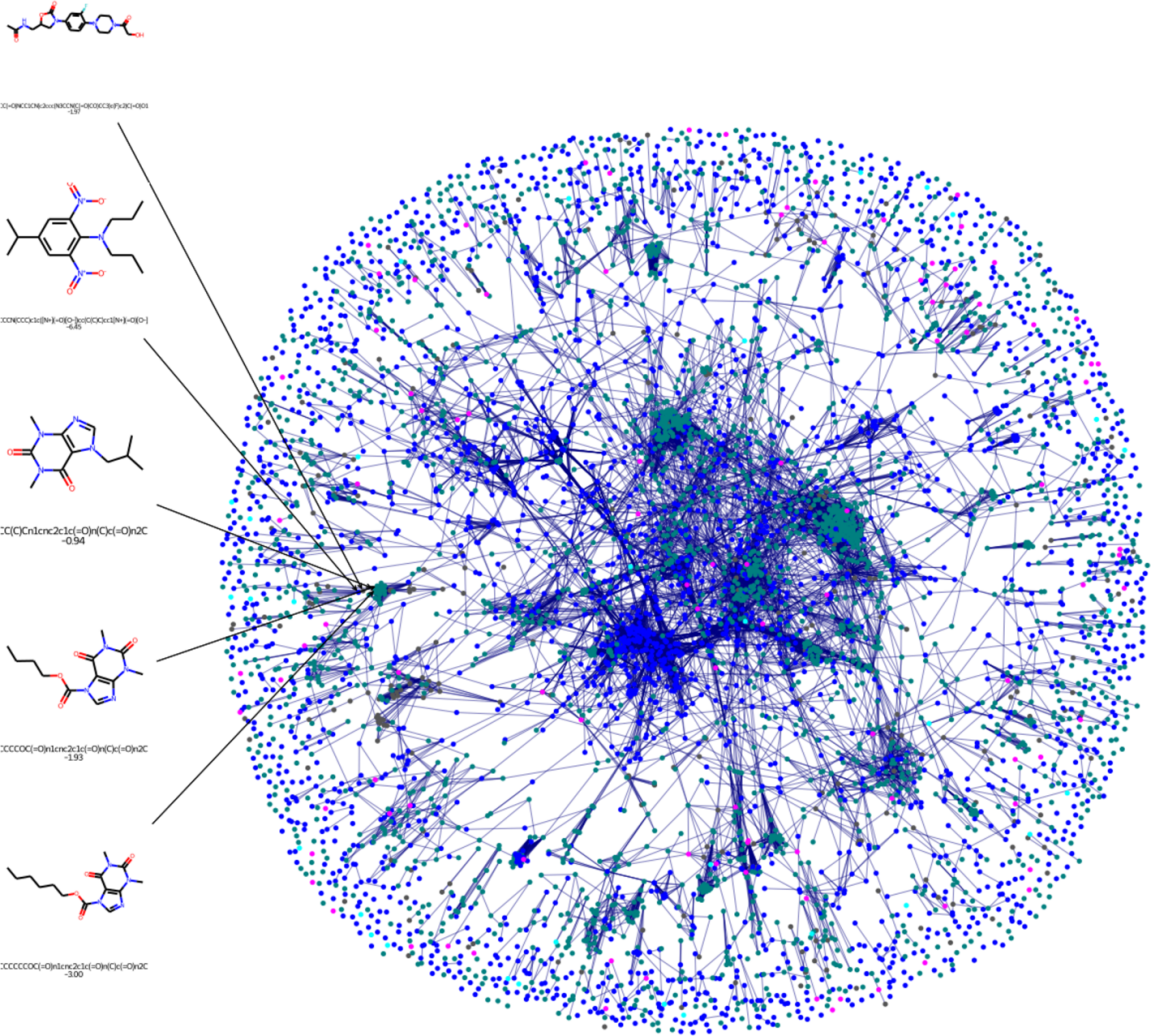
A graph representing the chemical space. Each node is one molecule, and node sizes are proportional to the number of connections (We note some points are hidden due to overlapping nodes in the tighter cluster.). D5697 are blue, D2999 are green, D300 are red, tight are pink, and loose are cyan. The graph topology comes from connecting nodes with a Tanimoto similarity of 0.5 and higher and running annealing through the Networkx implementation of the Fruchterman-Reingold force-directed algorithm.^48^

Figure 3 gives an additional interpretation of the chemical space of these data sets. It represents each molecule as a node in a graph, and the most similar (Tanimoto similarity scores of ≥0.5) are connected. The graph topology is generated through the Fruchterman-Reingold force-directed algorithm^48^ using Python’s NetworkX package (v.2.6.3). This algorithm treats the nodes as a set of spring connected particles and simulates the graph topology to a quasi-equilibrium state. In this case, the springs were weighted by the Tanimoto similarity score, making those nodes which have a higher Tanimoto similarity score relatively more attractive to one another. The nodes are colored by the data set. The pink and cyan colors are those associated with the tight and loose testing sets, respectively. We can see these nodes are distributed and typically connected to some nodes from the training sets. This again suggests that training data covers a chemical space inclusive of the space occupied by the testing data.

### Feature Sets

Features were calculated using structures generated from SMILES strings. Three different feature sets were generated using RDKit, Mordred,^49^ and Molecular Operating Environment (MOE) descriptors.^50^ Any descriptor which directly represented any definition of solubility was removed, in addition to any descriptor which had a Boolean or string output. For each feature set, any descriptor which returned an invalid output for any molecule was removed so that every molecule was described by the same set of valid descriptors. RDKit and Mordred features were generated using the Python API, while MOE descriptors were generated using MOE2018.01. It should be noted that Mordred is partially a Python wrapper of many available libraries, one of which is RDKit, and so the Mordred and RDKit feature sets have some common features, including the same calculated octanol–water partition coefficient (logP) descriptor.

#### RDKit

All descriptors available in the Crippen, Descriptors, Lipinksi, MolSurf, and QED modules were calculated. The resulting data set consisted of 205 exclusively 2D descriptors.

#### MOE

All available 2D and 3D descriptors were calculated. This resulted in a data set consisting of 359 descriptors, of which 229 were 2D and 130 were 3D. LigPrep^51^ was used to generate 3D conformations, and the lowest energy conformer was used to generate 3D descriptors.

#### Mordred

All available 2D descriptors were calculated. The resulting data set consisted of 972 exclusively 2D descriptors.

#### GNN

For the graph-based models, the default feature set for each model, based on DeepChem V2.4.0,^52^ was used. Atom features include element type, number of bonded neighbors, valence, charge, number of radical electrons and hybridization state. For the Weave model, additional features include the bond type, conjugation, and whether atoms are part of a ring. Chirality was ignored.

### Models

Three Random Forest (RF) models were developed using the RandomForestRegressor in Scikit-Learn V0.21.3,^53^ three neural networks (NN) were developed using the TensorFlow V1.13.1 implementation,^54^ and three different graph-based approaches (GNN) implemented in the DeepChem package^52^ V2.4.0 were tested. Each model was developed using Python V3.8.1 with key similarities, such as identical data set splits and validation method.

A nested cross-validation approach was used to train and validate the machine learning models. The outer cross-validation consisted of 50 resamples, each with a different randomly chosen 70%/30% train/test split. For each resample, parameters were tuned to minimize RMSE for 5-fold cross-validation on the training set, and then the selected parameters were used to retrain the model on the training set and predict the testing set. Hyperparameter optimization was performed using the in-built GridSearchCV function in SciKit-Learn for the RF models, and a custom-built grid search was developed and employed for the neural networks. The resulting testing set predictions were then averaged to obtain the validation results. The optimal hyperparameter combinations were then used to retrain the models on 100% of the training data before predictions were made on the tight and loose testing sets. The models are labeled such that the model type is shown with the feature set in superscript, where appropriate, with R referring to RDKit, MOE referring to MOE, and M referring to Mordred.

The NN models were built with the same general architecture of 3 densely connected hidden layers, each consisting of a smaller number of nodes compared to the previous layer. Each NN model used the ReLU activation in each layer and the Adam optimizer. The SciKit-Learn StandardScaler was employed on the feature sets to perform a mean-centered scaling of the features for use in the NN models.

A number of graph-based approaches were also tested using models available in the DeepChem package, namely the GraphConv,^55^ DAG (Directed Acyclic Graph),^56^ and Weave^57^ models. For all models, molecules are represented as graphs with each non-hydrogen atom represented by a feature vector. The Weave model also includes additional feature vectors for bonds. The custom-built grid search approach applied to the NN models was used to optimize the specific hyperparameters for the GraphConv and Weave models including batch size, learning rate, and the numbers and sizes of different layers within the networks. The default parameters were used for the DAG model. Models were trained for 200 epochs in total, and the model with the best performance on the validation set was retained.

### Statistical Analysis

To compare calculated and experimental results for different computational models, the coefficient of determination (*R*^2^) and the root mean squared error (RMSE) were evaluated
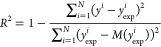
1
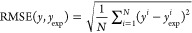
2where index *i* runs through the set of *N* selected molecules, and *y*^*i*^ and  are the calculated and experimental values of log(*S*), where *S* is given in molar units. The total deviation can be split into two parts: bias (or mean displacement, *M*) and standard deviation of the error of prediction (*SDEP*), which are calculated by the formulas
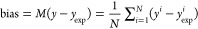
3
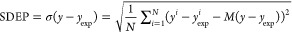
4The bias gives the systematic error, which can be corrected for in the final models by the addition of a simple constant term. The SDEP gives the random error that is not explained by the model. The connection with RMSE is given by
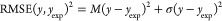
5Models reporting RMSE greater than the standard deviation of the experimental data offer less accurate predictions than the null model provided by the mean of the experimental data. Additionally, the percentage of predictions that were within ±0.5 log(*S*) of the experimental value (% ± 0.5 log(*S*)) was calculated to enable a comparison to results of the second solubility challenge.^29^

## Results and Discussion

### Validation

During the validation stage, machine learning models were trained and tested against the D300, D2999, and D5697 data sets. Table 1 shows the statistics for the models submitted to the solubility challenge as well as the RF^M^ model, each of which used the D300 training set, while Table 2 shows the statistics for the models trained on the D2999 and D5697 data sets. All of the statistics are reported as test set averages over 50 randomly selected training and test set splits. It is noted in passing that the models RF^R^ and RF^MOE^ were both submitted to the solubility challenge, with RF^MOE^ ranking within the top 10 submissions for prediction of the tight set. All other models were developed after the challenge had concluded.

**Table 1:** *R*^2^ and RMSE for Prediction of the Test Set Using RF Models and the D300 Data Set^a^

	D300	
model	*R*^2^	RMSE
RF^R^	0.61 ± 0.01	0.86 ± 0.01
RF^MOE^	0.64 ± 0.06	1.01 ± 0.12
RF^M^	0.69 ± 0.04	0.93 ± 0.09

a The statistics are reported as averages over 50 resamples (using 70%/30% train/test splits). Standard deviation of both *R*^2^ and RMSE is also shown.

**Table 2:** *R*^2^ and RMSE for Prediction of the Test Set Using Various Models and the D2999 or D5697 Data Set^a^

	D2999		D5697	
model	*R*^2^	RMSE	*R*^2^	RMSE
RF^R^	0.86 ± 0.01	0.84 ± 0.03	0.86 ± 0.01	0.86 ± 0.03
RF^M^	0.86 ± 0.01	0.84 ± 0.03	0.87 ± 0.01	0.85 ± 0.03
NN^R^	0.86 ± 0.01	0.86 ± 0.04	0.85 ± 0.01	0.89 ± 0.03
NN^M^	0.86 ± 0.01	0.86 ± 0.04	0.84 ± 0.01	0.88 ± 0.03
GraphConv	0.85 ± 0.01	0.87 ± 0.03	0.85 ± 0.01	0.89 ± 0.02
DAG	0.85 ± 0.01	0.88 ± 0.03	0.85 ± 0.01	0.88 ± 0.02
Weave	0.87 ± 0.01	0.82 ± 0.04	0.86 ± 0.01	0.85 ± 0.03

a The statistics are reported as averages over 50 resamples (using 70%/30% train/test splits). Standard deviation of both *R*^2^ and RMSE is also shown.

Some caution must be exercised in comparing the validation results for the D300, D2999, and D5697 data sets with each other because for each data set the resampled test sets are of different sizes and are drawn from different pools of molecules. Nonetheless, it is interesting to note the general trend that the predictive accuracy in terms of both *R*^2^ and RMSE increases when using the D2999 data set over D300, accompanied by a reduction in the statistical error. A further increase in the data set size to D5697 has little effect on the validation statistics or the statistical error. Each model was trained on random 70% splits of the training data; hence the larger data sets had not only more data to train on compared to D300 but also larger and potentially more diverse validation sets too. There are no results given for D300 using any neural network model as there was deemed to be an insufficient volume of data to reliably train these models. The extra data obtained in curating the larger data sets, D2999 and D5697, were necessary to regularize the neural networks and prevent them from overfitting. Note that there are no results given for models using MOE descriptors for D2999 and D5697 as access to the MOE software was lost between generating D300 and the larger data sets.

To understand how significantly each descriptor contributed to the performance of the RF models, we calculated the average Gini importance for the models trained on different data sets and descriptor sets (Figure S3 in the Supporting Information). In all cases, the models rely heavily on descriptors based on logP. The Mordred SLogP descriptor is a wrapper around the RDKit MolLogP descriptor, so these are identical for all molecules and are calculated from atomic contributions using the approach of Wildman and Crippen.^58^ The MOE descriptor set has a number of logP and logD based descriptors which contribute to the overall importance of logP. Other highly ranked features include molar refractivity and polar surface area and also descriptors which measure molecular size and complexity such as BertzCT; however, the importance scores for these descriptors are all significantly lower than for logP.

To analyze which molecular features the graph convolutional models had identified as having a key role in solubility, we generated counterfactual molecules using the procedure from Wellawatte et al.^59^ Counterfactuals were generated based on molecules in the D2999 training set by applying up to three mutations to the SELFIE representation of each molecule. These were then clustered based on fingerprint similarity and counterfactuals with a predicted solubility at least 1 log unit above or below the original molecule were selected. The RDKit fragment descriptors were used to identify the chemical features present in the original data set molecules and associated counterfactual molecules, and pairs of original and counterfactual molecules which differed in the value of just one of these descriptors were selected to analyze the effect of that change on the predicted solubility. For a given descriptor, the fraction (*f*) of all pairs of original and counterfactual molecules with an increase or decrease in solubility was calculated, and the difference is plotted in Figure S4 in the Supporting Information to show how strongly the chemical change represented by that descriptor is associated with an increase or decrease in predicted solubility. In most cases, the trends reflect chemical intuition, for example, increasing the number of hydrogen bond donors or acceptors, such as primary or secondary amines, leads to an increase in solubility, whereas addition of saturated hydrocarbon groups results in a lower predicted solubility.

### Solubility Challenge Data

The models were used to make predictions of both the tight and loose testing sets from the second solubility challenge. As described previously, the RF^R^ and RF^MOE^ models were submitted as blinded predictions during the challenge, and the remainder were developed and evaluated after the challenge finished. The prediction statistics calculated using the unblinded data after the conclusion of the solubility challenge are shown in Table 3. Since the tight set is larger than the loose set (100 molecules compared to 32 molecules), and the experimental error in the data is reported to be significantly lower (SD = 0.17 log units compared to SD = 0.62 log units), we will initially focus on the tight set results before discussing those for the loose set.

**Table 3:** *R*^2^, RMSE, SDEP, Bias, and % of Molecules Predicted within 0.5 Log Units of the True Solubility Value for Predictions on Both the Tight and Loose Testing Sets for Each of the Top Performing Models^a^

	tight set					loose set				
model	*R*^2^	RMSE	SDEP	bias	% ± 0.5 log	*R*^2^	RMSE	SDEP	bias	% ± 0.5 log
**D300**										
RF^R^	0.37	1.01	0.92	0.41	39	0.44	1.60	1.41	0.76	28
RF^MOE^	0.48	0.92	0.86	0.32	39	0.58	1.39	1.24	0.64	38
RF^M^	0.52	0.87	0.82	0.31	39	0.41	1.64	1.42	0.82	25
**D2999**										
RF^R^	0.44	0.94	0.85	0.42	44	0.60	1.36	1.17	0.69	28
RF^M^	0.51	0.89	0.79	0.39	45	0.54	1.45	1.26	0.72	34
NN^R^	0.35	1.02	0.98	0.28	37	0.57	1.40	1.29	0.55	34
NN^M^	0.54	0.86	0.73	0.45	53	0.54	1.45	1.20	0.81	25
GraphConv	0.48	0.91	0.78	0.48	48	0.23	1.88	1.44	1.21	22
DAG	0.29	1.06	0.96	0.46	43	0.36	1.71	1.45	0.90	28
Weave	0.54	0.86	0.77	0.38	55	0.62	1.32	1.16	0.63	31
**D5697**										
RF^R^	0.45	0.94	0.85	0.40	48	0.63	1.31	1.14	0.65	28
RF^M^	0.51	0.89	0.81	0.36	48	0.61	1.34	1.19	0.63	25
NN^R^	0.42	0.96	0.93	0.27	46	0.59	1.38	1.22	0.64	28
NN^M^	0.52	0.88	0.77	0.42	48	0.51	1.49	1.20	0.84	38
GraphConv	0.53	0.87	0.76	0.43	45	0.40	1.65	1.35	0.96	31
DAG	0.36	1.02	0.95	0.37	49	0.43	1.62	1.41	0.80	25
Weave	0.52	0.88	0.81	0.34	47	0.59	1.37	1.20	0.66	25

a Note that “bias” here is the mean-signed error, and *R*^2^ is the coefficient of determination.

Of the two models that were submitted to the solubility challenge, the Random Forest trained on MOE descriptors (RF^MOE^) was the most accurate on both the tight and loose data sets. This model was similar to one we published in 2007,^60^ but it had been retrained on the D300 data set using different software. By contrast, the Random Forest model trained on RDKit descriptors (RF^R^) performed less well on the tight and loose sets, even though it had been more accurate than RF^MOE^ during validation. Unlike the RF^MOE^ model, the RMSE for the RF^R^ predictions was significantly outside one standard deviation of the RMSE observed during validation. Taken alone at the time of the solubility challenge, this decrease in performance for the RDKit models was difficult to rationalize, but it does fit with trends observed in analyzing the models developed after the challenge finished, as described in more detail below.

Of the models developed after the solubility challenge finished, the best predictions of the tight set were obtained from the NN^M^ and Weave models trained on the D2999 data set, which were optimal in terms of *R*^2^ (0.54) and RMSE (0.86 log units), and predicted almost the same percentage of compounds within 0.5 log units of the true values (53% NN^M^, 55% Weave). Both models performed better than the RF^MOE^ model whose blinded predictions were submitted and ranked within the top 10 predictions of the tight set in the second solubility challenge.

**Figure 4:**
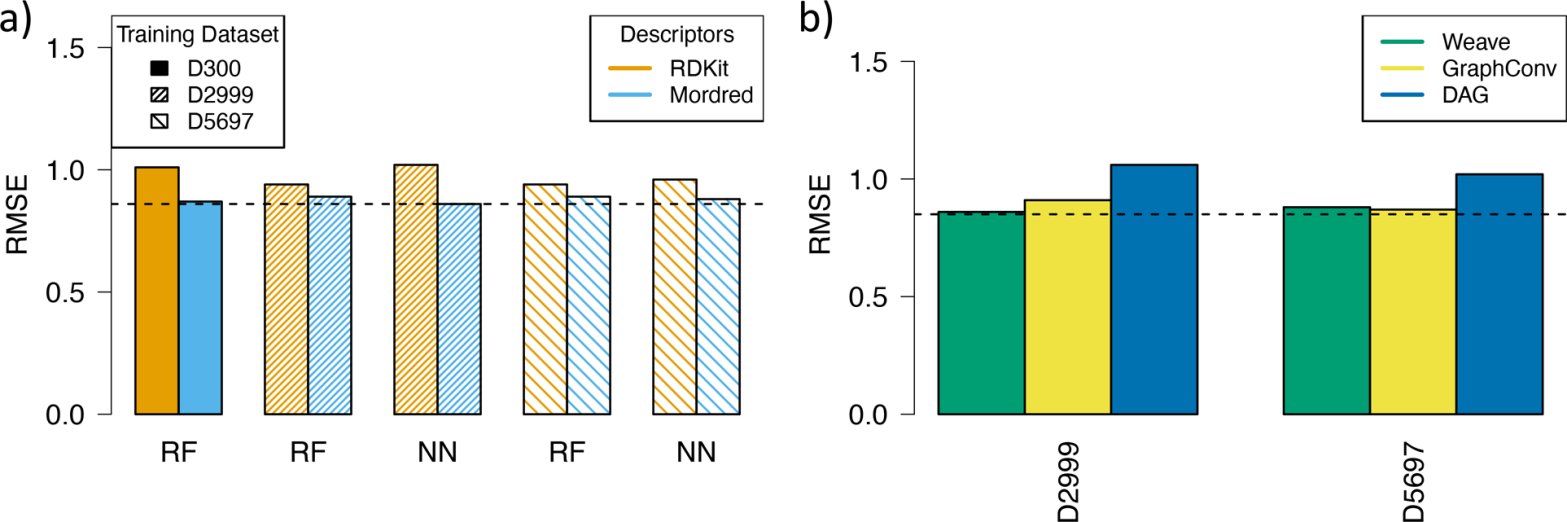
RMSE values for predictions of the tight set from the second solubility challenge: a) machine learning models built using Mordred or RDKit descriptors and b) three graph based deep learning models. The dotted horizontal line indicates the RMSE of the best model for reference (NN^M^, RMSE = 0.85 log units).

Among the traditional descriptor-based models, the choice of feature set had a larger influence on predictive accuracy than the choice of machine learning algorithm. Figure 4.a and Table 3 show that all of the models built with Mordred or MOE descriptors give similarly accurate RMSE values on the tight set, irrespective of the choice of machine learning algorithm, or training data set. However, the models trained using RDKit are consistently less accurate, which may indicate that the smaller RDKit feature set is missing some relevant information contained within the larger Mordred feature set. This trend fits with the observation noted earlier that the RDKit model (RF^R^) submitted to the solubility challenge performed less well than the MOE model (RF^MOE^) on the tight set.

Using graph convolutional neural networks gave predictions of similar accuracy to the best descriptor-based models but did not lead to a significant improvement, even when larger training data sets were employed. Figure 4.b shows that the Weave and GraphConv models predicted the tight set more accurately than the DAG model and that the performance of the Weave model was similar to the best machine learning model (NN^M^). The reason that the DAG model was less accurate is not clear. Although we were unable to complete a full search of the DAG hyperparameter space due to the computational cost of training the model, this does not seem to be the cause of the poorer tight set predictions, since the validation statistics show the model performing well and similarly to other methods.

**Figure 5:**
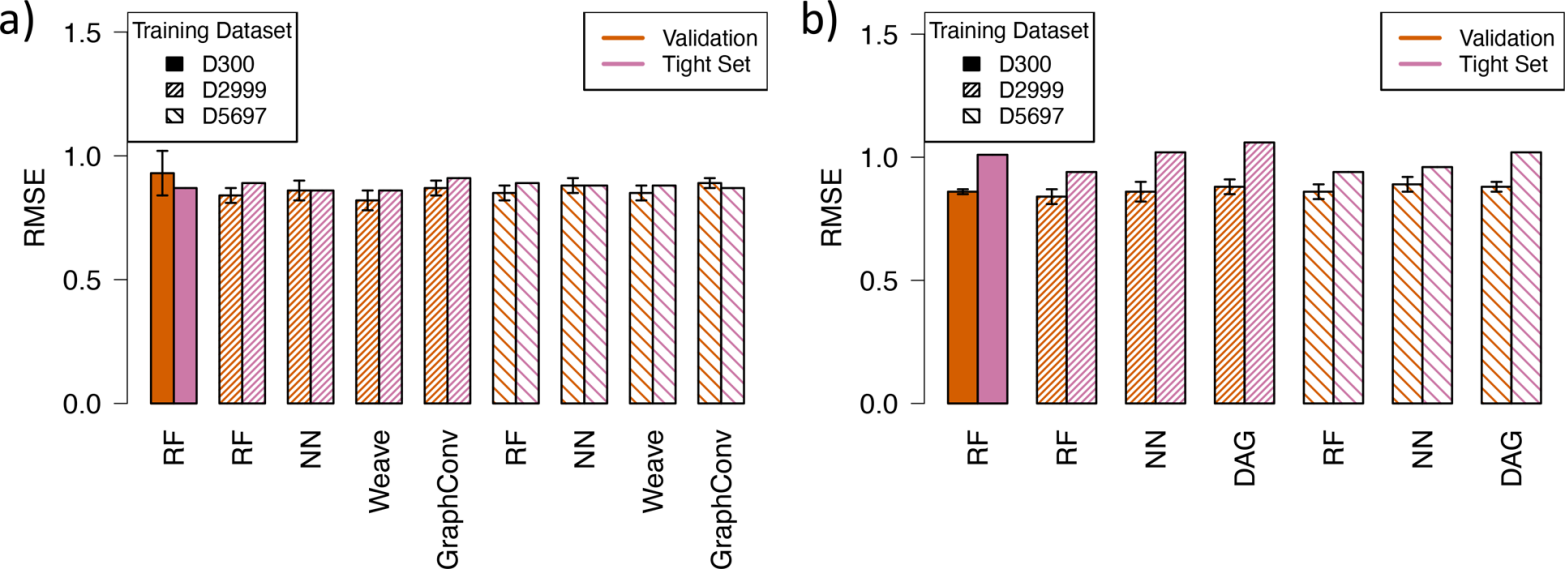
RMSE values for validation compared to RMSE values for prediction of the tight set from the second solubility challenge: a) models built using Mordred descriptors, Weave models, and GraphConv models and b) models built using RDKit descriptors and DAG models.

The experimental design used to train and validate the models (nested cross-validation) was successful in estimating the RMSE that would be obtained on the tight set by the best models but did not identify a group of models that performed less well, which is evident in comparing Figures 5.a and 5.b. For the models using Mordred and MOE descriptors and the Weave model, the testing set RMSE values are within one standard deviation of the corresponding validation RMSE, irrespective of the choice of the training data set or (where relevant) machine learning algorithm (Figure 5.a); the GNN models performed well too, but the testing set RMSE values are slightly outside one standard deviation in some cases. This means that the performance of these models on the tight set could have been accurately predicted from the validation data during model training. It gives confidence that these models are performing well since they have given consistent predictions on two separate data sets. By contrast, for the models using RDKit descriptors and the DAG models, the testing set RMSE values are noticeably worse than the corresponding validation RMSE, as demonstrated in Figure 5.b. Some possible reasons for the poorer performance of these models have already been discussed. Since the difference between the two groups of models would not have been predicted from the validation statistics alone, it suggests that the experimental design could be improved in future work, perhaps to include additional validation tests, such as leave-cluster-out, or other additional metrics to assess generalizability. These were not used here due to the time constraints imposed by the solubility challenge deadline.

**Figure 6:**
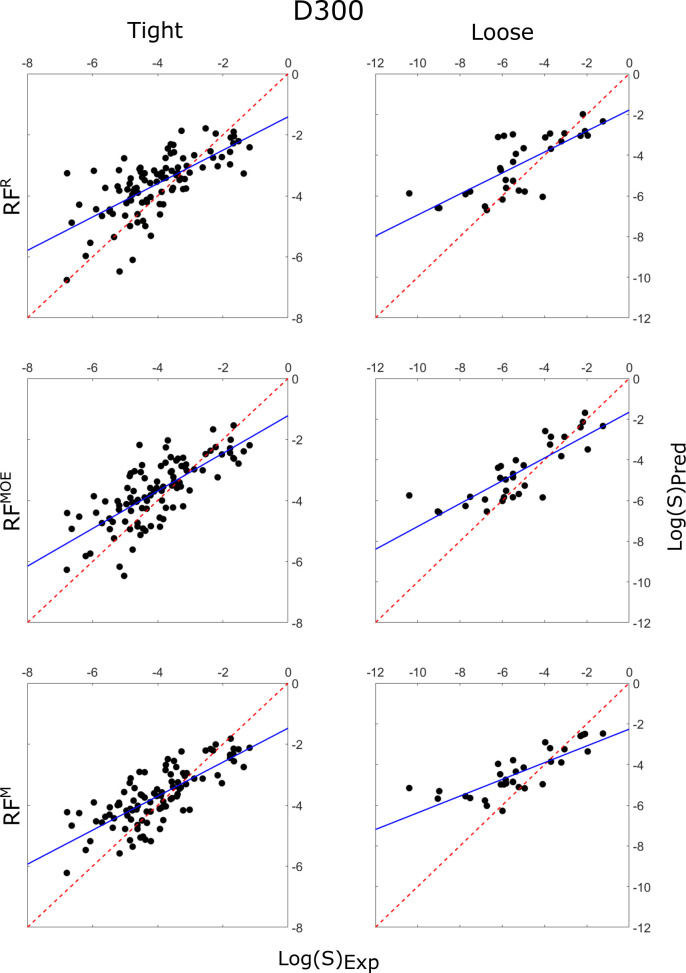
Correlation plots of the predicted intrinsic solubility values vs experimentally determined solubility values for RF^R^ (top), RF^MOE^ (middle), and RF^M^ (bottom), each predicting the tight (left) and loose (right) testing sets, using the D300 training set. The *y* = *x* line is plotted as a red dashed line, while the line of best fit is plotted as a solid blue line.

**Figure 7:**
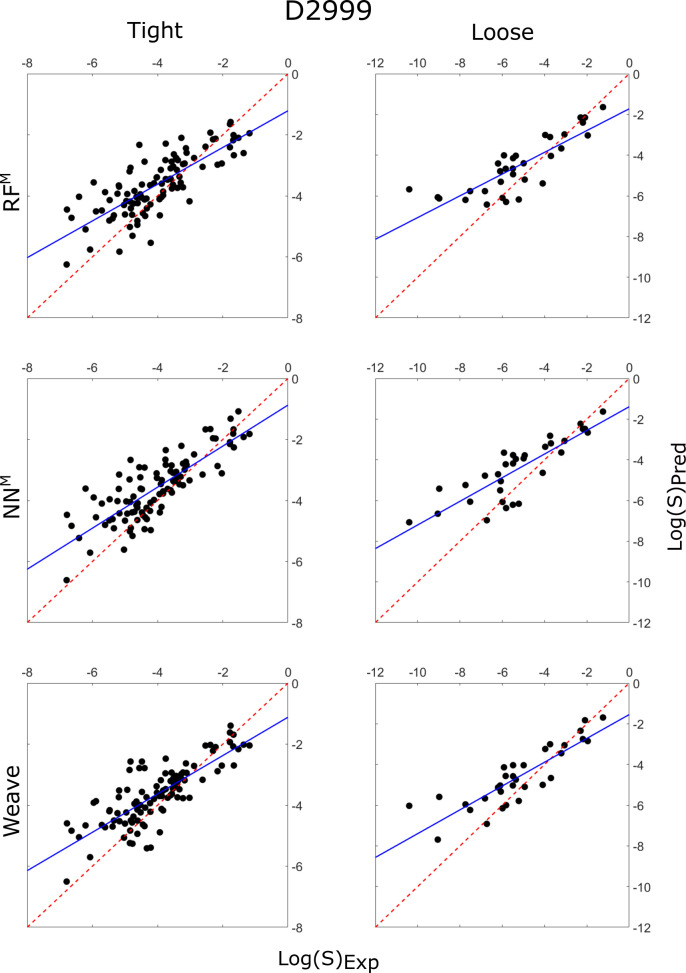
Correlation plots of the predicted intrinsic solubility values vs experimentally determined solubility values for RF^M^ (top), NN^M^ (middle), and Weave (bottom), each predicting the tight (left) and loose (right) testing sets, using the D2999 training set. The *y* = *x* line is plotted as a red dashed line, while the line of best fit is plotted as a solid blue line.

**Figure 8:**
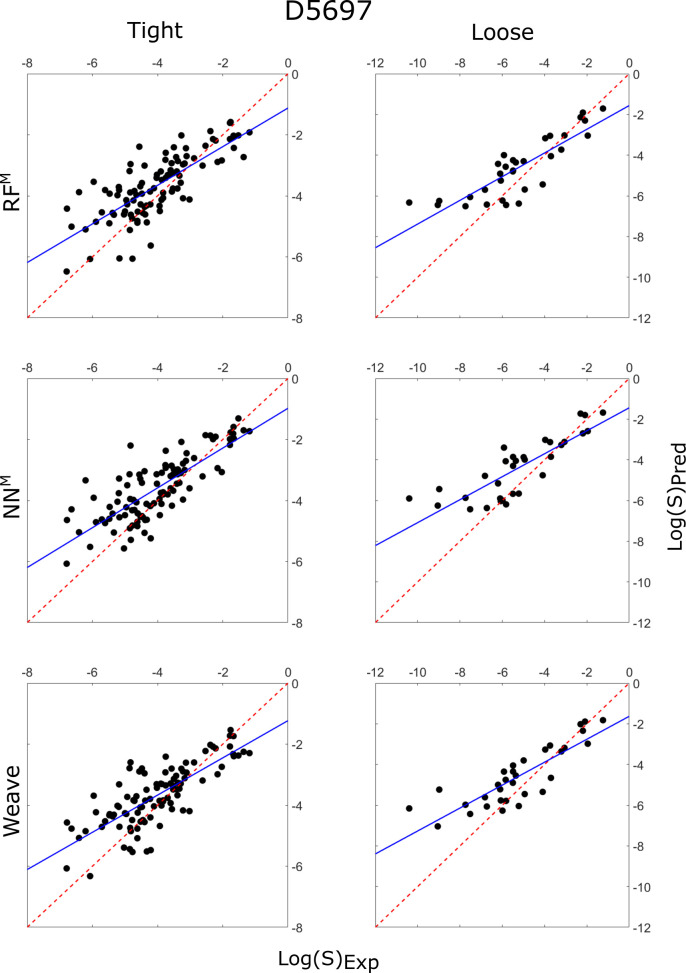
Correlation plots of the predicted intrinsic solubility values vs experimentally determined solubility values for RF^M^ (top), NN^M^ (middle), and Weave (bottom), each predicting the tight (left) and loose (right) testing sets, using the D5697 training set. The *y* = *x* line is plotted as a red dashed line, while the line of best fit is plotted as a solid blue line.

All of the models reported better predictions of the tight set than the loose set in terms of RMSE, bias, and number of molecules predicted within 0.5 log units of the true solubility (Table 3). The observation that the *R*^2^ is higher for the loose set can be explained by the larger range in experimental solubility data in the loose set (−10.4 < log(*S*) < −1.24) as compared to the tight set (−6.79 < log(*S*) < −1.18). Taken at face value, these statistics suggest that the low-variance tight set (σ ≈ 0.17) is easier to model than the higher-variance loose set (σ ≈ 0.62), which was the conclusion reached by Avdeef and Llinas in their summary of the findings of the second solubility challenge^29^ and by some other authors. However, it is interesting to note that the models presented here, and most of the other models submitted to the second solubility challenge, overpredict low solubility compounds and underpredict high solubility compounds (i.e., the gradient of the lines of best fit in Figures 6-8 are less than one). Since the same trend is evident in both the tight and loose sets, the range of experimental solubility data will affect all of the statistics used to compare the two data sets (not just *R*^2^). Considering the statistics in Table 4, which show the regression statistics for predictions of the 26 molecules in the loose set that have experimental solubilities within the range in the tight set (−6.79 < log(*S*) < −1.18), the picture is less clear. The best model (Weave/D2999) has a higher *R*^2^ and lower RMSE on this data set than it does on the tight set (*R*^2^ = 0.74 compared to *R*^2^ = 0.54, and RMSE = 0.80 log units compared to RMSE = 0.86 log units.), but a smaller percentage of the molecules are predicted within 0.5 log units (38.5% compared to 55%). In general, most of the models perform slightly better on the tight set than this data set, but the difference is much less pronounced than when the full range of experimental data in the loose set is considered. Increasing the representation of low solubility compounds in the training data might help to improve the predictions of the full loose set. Figure 1.b shows that the majority of training molecules have solubility values between 0 and −5 log units. Other than the low solubility compounds, whose solubilities were generally overpredicted as previously discussed, there were no molecules in the tight or loose sets that had consistently poor predictions across all models.

**Table 4:** *R*^2^, RMSE, SDEP, Bias, and % of Molecules Predicted within 0.5 Log Units of the True Solubility Value for Predictions on Those Molecules in the Loose Testing Set That Have Experimental Solubilities within the Range of Experimental Solubilities in the Tight Set (−6.79 < log *S* < −1.18)

	reduced loose set (*N* = 26)					loose set (*N* = 32)				
model	*R*^2^	RMSE	SDEP	bias	% ± 0.5 log	*R*^2^	RMSE	SDEP	bias	% ± 0.5 log
**D300**										
RF^R^	0.32	1.29	1.22	0.42	31	0.44	1.60	1.41	0.76	28
RF^MOE^	0.65	0.93	0.89	0.26	46	0.58	1.39	1.24	0.64	38
RF^M^	0.62	0.97	0.90	0.34	31	0.41	1.64	1.42	0.82	25
**D2999**										
RF^R^	0.64	0.94	0.88	0.35	35	0.60	1.36	1.17	0.69	28
RF^M^	0.66	0.91	0.85	0.31	42	0.54	1.45	1.26	0.72	34
NN^R^	0.56	1.03	1.02	0.15	42	0.57	1.40	1.29	0.55	34
NN^M^	0.61	0.98	0.90	0.40	31	0.54	1.45	1.20	0.81	25
GraphConv	0.37	1.24	1.01	0.72	27	0.23	1.88	1.44	1.21	22
DAG	0.57	1.03	0.94	0.41	35	0.36	1.71	1.45	0.90	28
Weave	0.74	0.80	0.76	0.26	39	0.62	1.32	1.16	0.63	31
**D5697**										
RF^R^	0.63	0.96	0.89	0.34	35	0.63	1.31	1.14	0.65	28
RF^M^	0.66	0.92	0.88	0.26	31	0.61	1.34	1.19	0.63	25
NN^R^	0.59	1.00	0.97	0.27	35	0.59	1.38	1.22	0.64	28
NN^M^	0.65	0.92	0.82	0.42	46	0.51	1.49	1.20	0.84	38
GraphConv	0.53	1.07	0.94	0.52	39	0.40	1.65	1.35	0.96	31
DAG	0.64	0.94	0.89	0.32	31	0.43	1.62	1.41	0.80	25
Weave	0.71	0.84	0.79	0.27	31	0.59	1.37	1.20	0.66	25

### Comparison to Other Methods

The results of the second solubility challenge reported by Llinas et al.^29^ show a wide range of prediction accuracy for the tight and loose sets. Of the 37 submissions to the challenge, the mean RMSE was 1.14 log units on the tight set and 1.62 log units on the loose set. Ignoring those submissions that had some overlap between the data used in training and the tight and loose testing sets, the best tight set predictions were provided by four models (three labeled as MLKC and one labeled as PMSA_A) which performed equally well with *R*^2^ and RMSE values of 0.60 and 0.80 log units, respectively. The MLKC submissions made use of lightGBM models with a feature set comprised of fingerprints and DRAGON features,^39^ while the PMSA_A submission used a radial basis function method coupled with an in-house feature set.^29^ The highest performing models reported in the present work (NN^M^ and Weave) achieved *R*^2^ = 0.54 and RMSE = 0.86 log units, placing them close to the highest performing submissions and improving upon our own best submission (JCSU_A, *R*^2^ = 0.48 and RMSE = 0.92 log units). The loose set was best predicted by the submission labeled UMUT_C, with *R*^2^ = 0.75 and RMSE = 1.06 log units. Of the models in this work, RF^R^ trained on the D5697 data set best predicted the loose set with *R*^2^ = 0.63 and RMSE = 1.31 log units, which was almost identical to the performance of the Weave model trained on the D2999 data set with *R*^2^ = 0.62 and RMSE = 1.32 log units. While this difference in performance between the best submission and our models is more significant than the tight set comparisons, the models presented here still perform better than most submissions to the challenge. Moreover, as discussed previously, several molecules with very low solubilities have a large effect on the predictive error of these models. Considering a solubility range comparable to the tight set, which is more representative of the range that is important for small organic molecules in practical applications for example within pharmaceutical drug discovery, the globally best model (Weave trained on the D2999 data set) gives *R*^2^ = 0.71 and RMSE = 0.80 log units, slightly improving on the statistics reported by the same model predicting the tight set.

### The Effect of Data Quality

Prior to the first solubility challenge, the error in published experimental data (which had been estimated to be approximately 0.6 log units) was often cited as the limiting factor preventing solubility models from improving. The implicit hypothesis was that training and testing solubility models on more accurate data would lead to more accurate predictions, but this was not borne out by the results of the first solubility challenge, where the best models reported RMSE of 0.7–1.1 log units even though the experimental error in the solubility data was reported to be close to 0.05 log units. [Later studies estimated the experimental error to be approximately 4-fold higher but still significantly lower than the predictive error of the best solubility models.] In 2015, we reported a direct comparison of QSPR models developed on the same set of molecules but different sources of experimental data from which we concluded that “it is the deficiency of QSPR methods (algorithms and/or descriptor sets), and not, as is commonly quoted, the uncertainty in the experimental measurements, which is the limiting factor in accurately predicting aqueous solubility for pharmaceutical molecules”.^15^ One of the stated aims of the second solubility challenge was to revisit that conclusion by incorporating both a low-variance tight set (n = 100, σ ≈ 0.17 log units) and a high-variance loose set (n = 32, σ ≈ 0.62 log units). However, since corresponding low- and high-variance training sets were not provided with the second solubility challenge, interpretation of the previously published results is not straightforward. Here, we constructed three training data sets of differing sizes, which reflected a trade-off between the reliability of experimental data and the number of data points, and this consequently affected the coverage of chemical space too. Increasing the size of the data set from 300 molecules to 2999 molecules slightly improved the predictions on the tight and loose sets, but further expanding it to 5697 molecules had a negligible effect, even for the deep learning models which would have been expected to benefit most from the extra data. This observation may be partially explained by the volume and quality of data for training. While the experimental data in the D300 set is reasonably accurate, it has too few data points to reliably train the more complicated machine learning or deep learning algorithms. Conversely, the D5697 data set contains too much experimental solubility data of unknown provenance. However, it is likely that the coverage of chemical space is also important. Many of the extra molecules included in the D5697 data set come from a region of chemical space that is dissimilar to the tight or loose sets. Indeed, although the D5697 data set contains significantly more unique Murcko scaffolds (1222 compared to 798 for D2999 or 142 for D300), the number of Murcko scaffolds in common with either the tight or loose set remains approximately constant (38, as compared to 37 for D2999 or 21 for D300. See Table S2 in the Supporting Information). While the extra data in the D5697 data set may have helped to regularize the neural networks, it did not significantly increase the relevant information content of the training data and therefore did not lead to improved predictions.

## Conclusions

The models presented in this work have been shown to perform to a high standard, producing statistics comparable to the highest performing submissions to the second solubility challenge, and to other more recent developments, with the highest performing neural network model yielding an *R*^2^ of 0.54 and RMSE of 0.86 log units for the tight set. While these results are promising, further improvements may be made by additional refinement of the neural networks or possibly by improved training data selection. The volume of available training data has a notable effect on the predictive accuracy of machine learning models, with a higher volume increasing performance, as long as the additional data is of good quality and from a relevant region of chemical space. Comparison of the results using the three differently sized training sets emphasizes this point: D2999 increased model performance compared to D300, while D5697 did not result in an increase in performance as the additional data was of lower reliability and did not significantly increase the representation of the relevant regions of chemical space. Part of the difficulty in developing general solubility models is in representing a diverse chemical space from the relatively small number of consistent solubility measurements in the published literature. In focused practical applications, such as lead discovery or lead optimization, where data is collected in a consistent manner and the structures and data range of the training set are likely to be consistent with those of future synthesized compounds, building bespoke models on the scaffolds of interest is likely to be important. In developing general solubility models from literature data, as was necessary for the second solubility challenge, data set selection remains a trade-off between data set size and data quality; including more data may be beneficial during training but usually means accepting more lower quality data. Moreover, in practice, when data is limited, it may mean accepting more data from less relevant regions of chemical space. Additionally, the majority of available experimental solubility data lies within a more narrow range than some of the compounds in the test sets, so training and prediction on molecules with more extreme low or high solubilities may be limited by a lack of appropriate data. Measurement and curation of larger consistent experimental data sets will benefit solubility prediction in the future, but other factors such as the failure of models to differentiate between crystalline polymorphs or to interpolate accurately between sparse data points must also be addressed to improve prediction accuracy.

## Data and Software Availability

The solubility data sets are available in comma separated variable format in the Supporting Information; these files include experimental solubility measurements, molecular structures as SMILES strings, and molecular descriptors. The code to train the selected machine learning models is available at DOI 10.5281/zenodo.7130065.

## Supporting Information Available

Solubility data sets (CSV) including experimental solubility measurements, molecular structures as SMILES strings, and molecular descriptors (ZIP)
Figure S1, kernel density plot; Table S1, summary statistics; similarity analysis; Figure S2, percentage of compounds from each data set which have at least one similar compound in each of the other data sets; Table S2, number of unique Murcko molecular scaffolds per data set; Figure S3, feature importance for top 15 descriptors from RF models; Table S3, list of hyperparameters optimized in NN models; Table S4: list of hyperparameters optimized in graph convolutional neural network models; Figure S4, number of pairs of molecules between training set and generated counterfactuals and difference in fraction of pairs of molecules; and Figure S5, PCA and t-SNE plots of three training data sets (PDF)
