# Supplementary material for: Blinded Predictions and Post Hoc Analysis of the Second Solubility Challenge Data: Exploring Training Data and Feature Set Selection for Machine and Deep Learning Models
Source: J Chem Inf Model. 2023 Feb 9;63(4):1099–113. doi: 10.1021/acs.jcim.2c01189 (PMC9976279; doi:10.1021/acs.jcim.2c01189)
Supplement: Supplementary file 2 — ci2c01189_si_002.pdf [file ci2c01189_si_002.pdf]

Supporting Information: "Blinded Predictions and Post-hoc Analysis of the Second Solubility Challenge Data: Exploring Training Data and Feature Set Selection for Machine and Deep Learning Models"

Jonathan G. M. Conn,[†] James W. Carter,[†] Justin J. A. Conn,[†] Vigneshwari Subramanian[1],[‡] Andrew Baxter,[¶] Ola Engkvist,[§,‖] Antonio Llinas,[‡] Ekaterina L. Ratkova,[§] Stephen D. Pickett,[⊥] James L. McDonagh,[#] and David S. Palmer[*,†]

†*Department of Pure and Applied Chemistry, University of Strathclyde, Thomas Graham Building, 295 Cathedral Street, Glasgow G1 1XL, U.K.*

‡*Drug Metabolism and Pharmacokinetics, Research and Early Development, Respiratory & Immunology, BioPharmaceuticals R&D, AstraZeneca, Pepparedsleden 1, SE-431 83 Göteborg, Sweden*

¶*GSK Medicines Research Centre, Gunnels Wood Road, Stevenage, SG1 2NY, U.K.*

§*Medicinal Chemistry, Research and Early Development, Cardiovascular, Renal and Metabolism (CVRM), BioPharmaceuticals R&D, AstraZeneca, SE-431 50 Göteborg, Sweden*

‖*Department of Computer Science and Engineering, Chalmers University of Technology, SE-412 96 Göteborg, Sweden*

⊥*Computational Chemistry, GlaxoSmithKline R&D Pharmaceuticals, Stevenage SG1 2NY, U.K.*

#*IBM Research Europe, Hartree Centre, SciTech Daresbury, Warrington, Cheshire WA4 4AD, U.K.*

E-mail: david.palmer@strath.ac.uk

[1] *Current Address: Imaging and Data Analytics, Clinical Pharmacology & Safety Sciences, R&D, AstraZeneca, Pepparedsleden 1, SE-431 83 Göteborg, Sweden*

Experimental Data

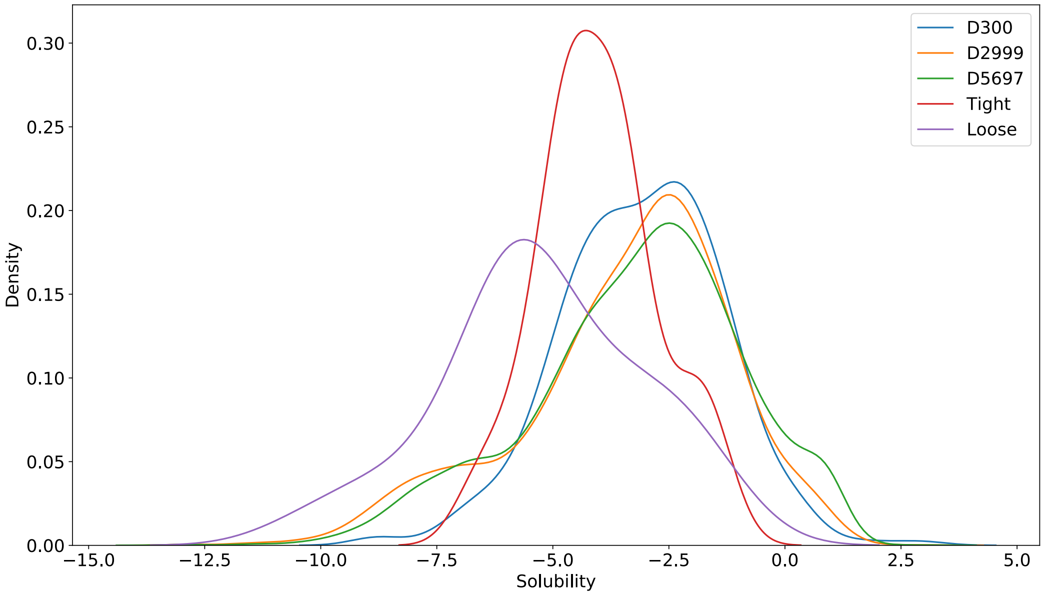

Figure S1: Kernel density plot showing the ranges of solubility values in the three training datasets (D300, D2999, and D5697), and the tight and loose sets from the Second Solubility Challenge.

Table S1: Summary statistics comparing the distributions of experimental solubility data (as $Log_{10}S$, where S is in molar units), molecular weight, and the number of rotatable bonds per molecule, for each of the three training datasets (D300, D2999, and D5697) and the tight and loose sets from the Second Solubility Challenge.

**Dataset**	**No. of mols**	**Log(S)**			**Mol. Weight**			**No. Rotatable Bonds**		
		**Min**	**Max**	**Mean**	**Min**	**Max**	**Mean**	**Min**	**Max**	**Mean**
D300	300	-8.8	2.89	-3.08	89.05	776.69	273.73	0	14	2.98
D2999	2999	-12.95	2.89	-3.42	59.04	1296.42	266.27	0	20	2.94
D5697	5697	-13.17	2.89	-3.20	50.02	1296.42	246.06	0	20	3.43
Tight	100	-6.79	-1.18	-4.03	152.03	1201.84	338.33	0	17	4.08
Loose	32	-10.4	-1.24	-5.24	151.14	846.44	408.37	0	12	5.4

Similarity Analysis

The percentage of compounds in each dataset which have a Tanimoto similarity of more than 0.35 to at least one compound in the other datasets was calculated to illustrate the

similarity between datasets. All of the compounds in the tight and loose test sets have at least one similar compound in each of the training sets, based on this similarity threshold.

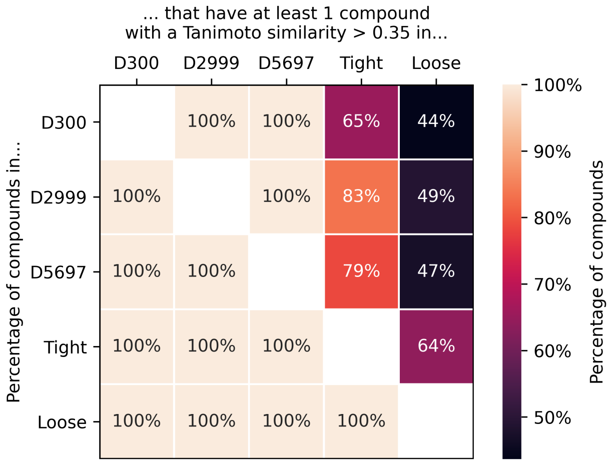

Figure S2: Percentage of compounds from each dataset which have at least one similar (Tanimoto similarity $>$ 0.35) compound in each of the other datasets.

Scaffold Analysis

Table S2: Number of unique Murcko molecular scaffolds per dataset, and the number in common between each training dataset and either the tight or loose test set.

Dataset	Number of Murcko Scaffolds	Number of Murcko Scaffolds in common with Tight Set	Number of Murcko Scaffolds in common with Loose Set
Tight	85		
Loose	30		
D300	142	18	3
D2999	798	30	7
D5697	1222	31	7

Feature importance

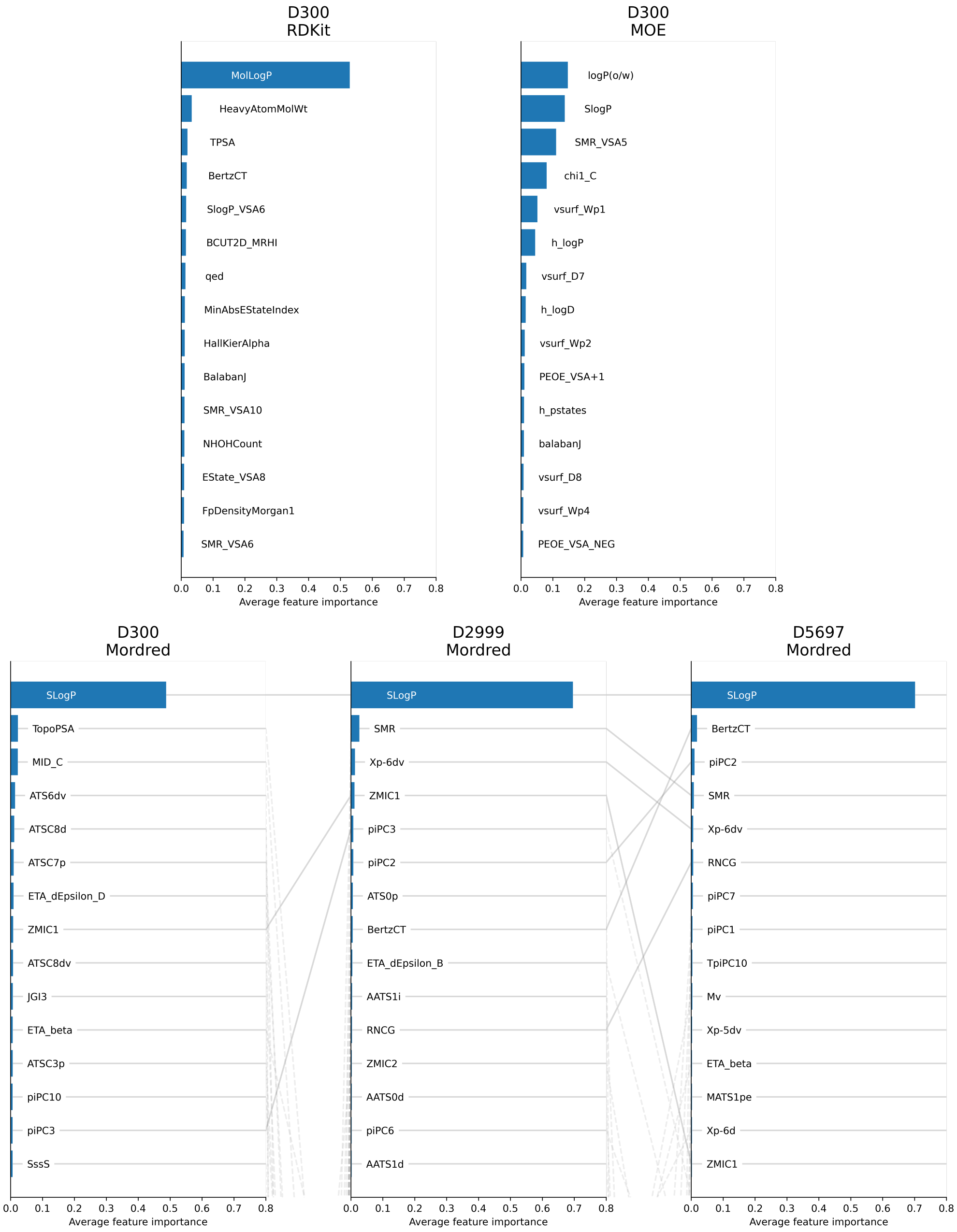

Figure S3: Feature importance for the top 15 descriptors from RF models trained on different datasets and descriptors and averaged over 50 resamples. On the bottom panel the same Mordred descriptors are joined by grey lines to highlight the relative rankings of these descriptors in models trained on the three different datasets. Dashed lines indicate connections to descriptors not in the top 15.

To understand how significantly each descriptor contributes to the performance of the RF models, we calculate the average Gini importance for the models trained on different datasets and descriptor sets and these are plotted in figure S3. In all cases the models rely heavily on descriptors based on logP. The Mordred SLogP descriptor is a wrapper around the RDKit MolLogP descriptor so these are identical for all molecules and are calculated from atomic contributions using the approach of Wildman and Crippen.[1] The MOE descriptor set has a number of logP and logD based descriptors which contribute to the overall importance of logP. Other highly ranked features include molar refractivity and polar surface area and also descriptors which measure molecular size and complexity such as BertzCT, however, the importance scores for these descriptors are all significantly lower than for logP. In the bottom panel of figure S3 we compare the ranking of Mordred descriptors in RF models trained using the three different training datasets. In all cases logP is clearly the most significant descriptor. Below this, there is little overlap between the top ranking descriptors in models trained on the D300 and D2999 sets indicating that the choice of additional descriptors is strongly tailored to the training sets. However, there is more overlap between the top ranking descriptors in the D2999 and D5697 sets, reflecting the higher degree of overlap between these datasets with the D2999 dataset representing over half of the data in the D5697 set.

Neural Network Hyperparameters

Table S3: List of hyperparameters optimised in the NN models. Entries in rounded brackets denote a range of values where (a, b, c) shows initial value of a, increasing to b in increments of c. Entries in square brackets denote a vector of values. "HL" refers to the hidden layers.

**Hyperparameter**	**Model**		
	**NN**^{R}	**NN**^{MOE}	**NN**^{M}
HL1 nodes	(50, 150, 10)	(100, 300, 20)	(300, 700, 50)
HL2 nodes	[25, 50, 75]	[50, 100, 150]	[150, 250, 350]
HL3 nodes	[15, 30, 45]	[25, 50, 75]	[75, 125, 175]
Batch size	[16, 32]	[16, 32]	[16, 32]
Learning rate	[10^{-3}, 10^{-4}, 10^{-5}]	[10^{-3}, 10^{-4}, 10^{-5}]	[10^{-3}, 10^{-4}, 10^{-5}]
Loss function	[mae, mse]	[mae, mse]	[mae, mse]

Table S3 shows the hyperparameters used in the optimisation of the neural networks. The values/options for batch size, learning rate, and loss functions which were searched over for optimisation were kept identical between each feature set. The range of values over which the hidden layer sizes were optimised were not kept identical across feature sets as the number of input layer nodes is equal to the feature set size. Hence, each network was optimised according to the number of features that were input to the model.

Table S4: List of hyperparameters optimised in the graph convolutional neural network models. Entries in square brackets denote a vector of values.

**Hyperparameter**	**Model**	
	**GraphConv**	**Weave**
graph_conv_layers	[[64], [256], [64, 64], [64, 128], [128, 64]]	-
dense_layer_size	[128, 256, 64]	-
n_hidden	-	[50, 25, 100]
fully_connected_layer_sizes	-	[[2000], [2000, 100], [2000, 1000, 100]]
dropout	[0, 0.1]	
Batch size	[50, 100]	
Learning rate	10^{-3}	
Loss function	mse	

Model explainability using counterfactuals

To analyse which molecular features the graph convolutional model trained on the D2999 dataset has identified as having a key role in solubility we generate counterfactual molecules using the procedure from Wellawatte *et. al.*[2] Counterfactuals were generated based on molecules in the D2999 training set by applying up to three mutations to the SELFIE representation of each molecule. These were then clustered based on fingerprint similarity and counterfactuals with a predicted solubility at least 1 log unit above or below the original molecule were selected. The RDKit fragment descriptors were used to identify the chemical features present in the original dataset molecules and associated counterfactual molecules, and pairs of original and counterfactual molecules which differed in the value of just one of these descriptors were selected to analyse the effect of that change on the predicted solubility. For a given descriptor, the fraction (f) of all pairs of original and counterfactual molecules with an increase or decrease in solubility was calculated and the difference is plotted in Fig. S4 to show how strongly the chemical change represented by that descriptor is associated with an increase or decrease in predicted solubility. The descriptors are ordered based on the effect on solubility from functional groups which reduce solubility on the left-hand side to groups which increase solubility on the right-hand side. In most cases this reflects chemical intuition, for example, increasing the number of hydrogen bond donors or acceptors, such as primary or secondary amines, leads to an increase in solubility, whereas addition of saturated hydrocarbon groups results in a lower predicted solubility.

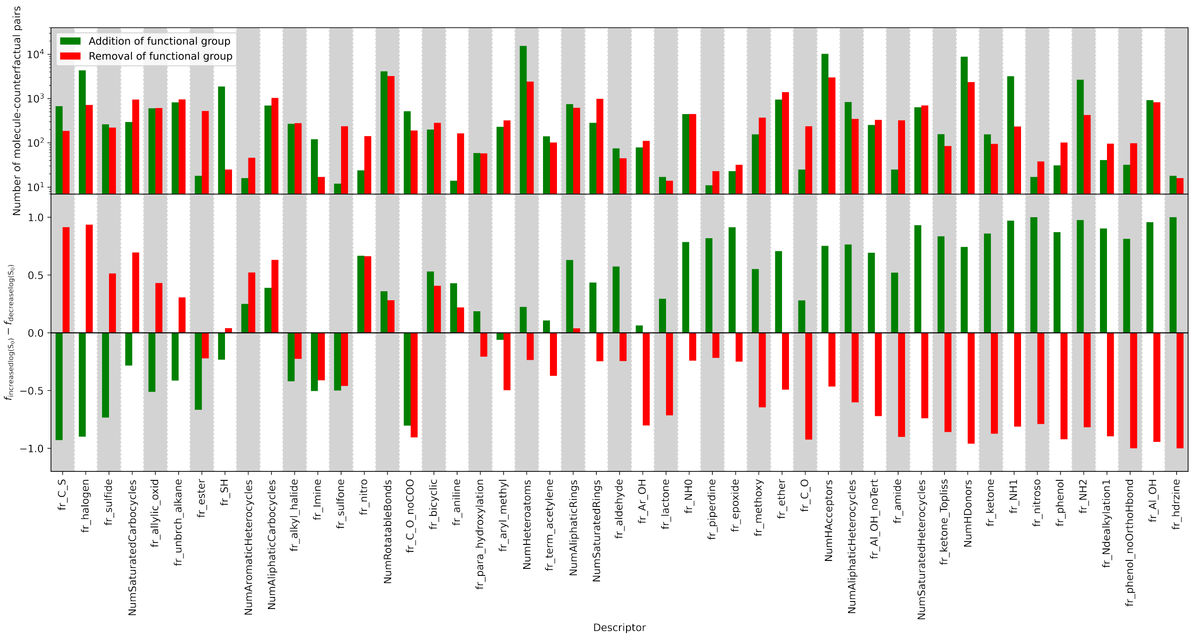

Figure S4: Top) Number of pairs of molecules between the training set and generated counterfactuals which differ only in a single fragment descriptor. Bottom) The difference in the fraction of these pairs of molecules which have an increase or decrease in the predicted solubility.

PCA and t-SNE Plots

PCA was performed on the entire dataset, combining the full training set (D5697) and the tight and loose test sets, with molecules represented using either RDKit fingerprints, Mordred descriptors or the neural fingerprints output by the GraphConv model trained on the D5697 dataset. In figure S5-top, the first 2 principle components are plotted for each molecular representation, with each training dataset plotted separately to highlight differences in the relative coverage of chemical space. t-SNE plots were calculated using the first 50 principle components for each molecular representation and the results are plotted in figure S5-bottom, mirroring the arrangement of the PCA plots above.

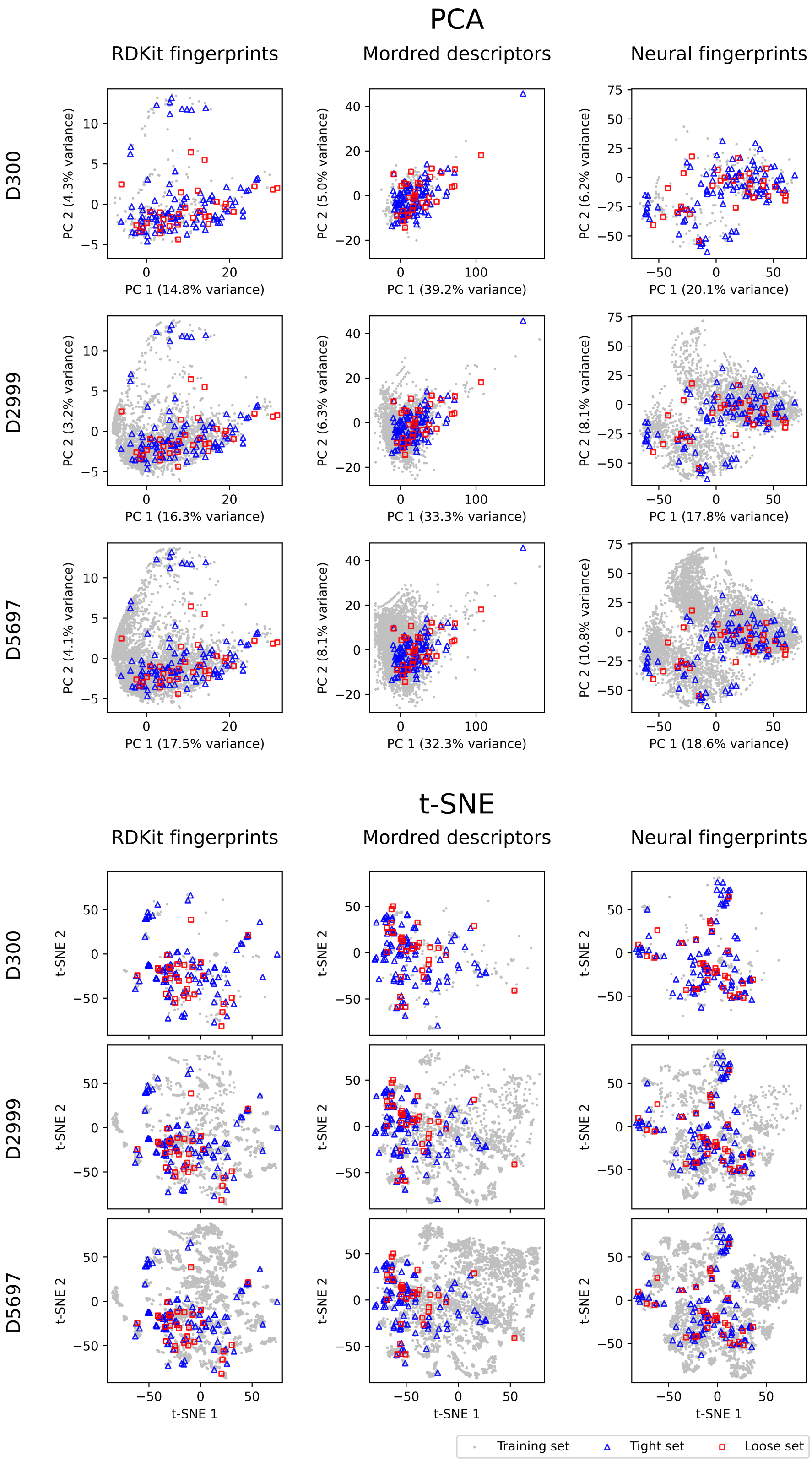

Figure S5: PCA (top) and t-SNE (bottom) plots of the three training datasets represented using RDKit fingerprint (left column), Mordred descriptors (middle column) and neural fingerprints from the GraphConv model trained on the D5697 dataset (right column).

Bibliography

(1) Wildman, S. A.; Crippen, G. M. Prediction of Physicochemical Parameters by Atomic Contributions. *J. Chem. Inf. Comput. Sci.* **1999**, *39*, 868–873.

(2) Wellawatte, G. P.; Seshadri, A.; White, A. D. Model agnostic generation of counterfactual explanations for molecules. *Chem. Sci.* **2022**, *13*, 3697–3705.
